# *In vitro* study on the feasibility of magnetic stent hyperthermia for the treatment of cardiovascular restenosis

**DOI:** 10.3892/etm.2013.1177

**Published:** 2013-06-21

**Authors:** LI LI, RUI WANG, HUAN-HUAN SHI, LE XIE, JING-DING-SHA LI, WEI-CHAO KONG, JIN-TIAN TANG, DA-NIAN KE, LING-YUN ZHAO

**Affiliations:** 1 Department of Oncology, Xiangya Hospital, Central South University, Changsha, Hunan 410008;; 2 Institute of Medical Physics and Engineering, Key Laboratory of Particle & Radiation Imaging, Ministry of Education, Department of Engineering Physics, Tsinghua University, Beijing 100084;; 3 Harbin Stomatology Hospital, Harbin, Heilongjiang 150010;; 4 Department of Bio-Pharmaceutics, Beijing University of Chinese Medicine, Beijing 100102;; 5 Beijing MED Zenith Medical Scientific Co. Ltd, Beijing 101312;; 6 Graduate School, Hunan University of Chinese Medicine, Changsha, Hunan 410208, P.R. China

**Keywords:** magnetic stent hyperthermia, percutaneous transluminal coronary angioplasty, restenosis, vascular smooth muscle cells, alternative magnetic field

## Abstract

Thermal treatment or hyperthermia has received considerable attention in recent years due to its high efficiency, safety and relatively few side-effects. In this study, we investigated whether it was possible to utilize targeted thermal or instent thermal treatments for the treatment of restenosis following percutaneous transluminal coronary angioplasty (PTCA) through magnetic stent hyperthermia (MSH). A 316L stainless steel stent and rabbit vascular smooth muscle cells (VSMCs) were used in the present study, in which the inductive heating characteristics of the stent under alternative magnetic field (AMF) exposure, as well as the effect of MSH on the proliferation, apoptosis, cell cycle and proliferating cell nuclear antigen (PCNA) expression of the rabbit VSMCs, were evaluated. The results demonstrated that 316L stainless steel coronary stents possess ideal inductive heating characteristics under 300 kHz AMF exposure. The heating properties were shown to be affected by the field intensity of the AMF, as well as the orientation the stent axis. MSH had a significant effect on the proliferation and apoptosis of VSMCs, and the effect was temperature-dependent. While a mild temperature of 43°C demonstrated negligible effects on the growth of VSMCs, MSH treatment above 47°C effectively inhibited the VSMC proliferation and induced apoptosis. Furthermore, a 47°C treatment exhibited a significant and long-term inhibitory effect on VSMC migration. The results strongly suggested that MSH may be potentially applied in the clinic as an alternative approach for the prevention and treatment of restenosis.

## Introduction

Coronary atherosclerosis, the hardening of arteries due to a build-up of lipoproteins, is one of the leading causes of mortality in many countries (1,2). Since percutaneous transluminal angioplasty (PTCA) was introduced by Grüntzig *et al* (3), it has rapidly become the most frequently applied interventional therapy for heart attacks and has been recommended as the standard of care (4). Following the introduction of PTCA, the combinatorial method of metallic stent implantation along with angioplasty was developed, in order to improve the efficacy of the coronary angioplasty and to ensure that the treatment provided a permanent solution for an occluded artery. However, restenosis following angioplasty, with or without stent implantation, occurs in 35–45% of patients at 6 months, and represents one of the most critical problems with this technique (5). Although the molecular mechanisms of restenosis are poorly understood, and numerous factors, including inflammation, granulation and extracellular matrix remodeling, may be involved in the process of restenosis, it has been elucidated that the abnormal proliferation and migration of vascular smooth muscle cells (VSMCs) in the neointima is one of the main causes of restenosis following angioplasty and stent implantation (6).

In order to prevent and treat restenosis, novel devices and protocols, such as drug-eluting stents (DESs), gene therapy, brachytherapy and laser treatment, have been developed (7–10). The stent-based local release of antiproliferative agents, such as sirolimus or paclitaxel, at the site of vascular injury via polymer-coated stents has been shown to result in effective local drug concentrations for a designated period, and to avoid systemic toxicity (11). Advances in DESs have substantially reduced the incidence of restenosis; however, there has been little impact on the long-term prognosis as compared with bare metal stents (BMSs) (12). Furthermore, there are considerations regarding the incidence of instent thrombosis and safety concerns with DESs. Therefore, alternative medical treatments for the inhibition of VSMC proliferation are required.

Recently, the advantages of thermal treatment have been recognized. As an effective, safe and environmentally sound approach, thermal treatment or hyperthermia has been applied as a monotherapy and as an adjunctive therapy (13). Although hyperthermia has predominantly been utilized for the treatment of cancer, the clinical applications of hyperthermia are gradually being extended, indicating that the physiological effects of heating treatments may be wide-ranging. It has been revealed that repeated whole-body hyperthermia may improve vascular endothelial and cardiac functions in patients with chronic heart failure (14). Furthermore, Orihara *et al* showed that hyperthermia treatment (43°C, 2 h) was capable of inhibiting the proliferation of the dividing VSMCs without damaging the quiescent VSMCs (15). However, with regard to restenosis, which normally occurs at the site of the stent in the coronary artery, there is a requirement for localized or targeted heating. The coronary stents developed for clinical application are most commonly made of a metal alloy, such as 316L stainless steel, nickel-titanium (Ni-Ti) or cobalt-chrome (Co-Cr), which demonstrate the desired inductive heating characteristics under an alternative magnetic field (AMF). It is thus feasible that localized heating through magnetic stent hyperthermia (MSH) may be possible.

In this study, we investigated the inductive heating characteristics of the stents that are currently utilized in clinical application under AMF exposure. Rabbit VSMCs were used to study the effect of MSH on the cell cycle, cell apoptosis and the expression of proliferating cell nuclear antigen (PCNA). The results are likely provide useful information to aid the understanding of the mechanisms behind the effects of heat treatments and AMF exposure on smooth muscle cells. The deductions from the experimental conclusions may have significance with regard to the use of MSH as an alternative approach for the treatment of restenosis.

## Materials and methods

### Cell culture

In the present study, rabbit VSMCs were provided by the Cell Center of the Institute of Basic Medical Sciences, Chinese Academy of Medical Sciences and Peking Union Medical College (Beijing, China). Cells between passages four and ten were used. The VSMCs were cultured in Dulbecco’s modified Eagle’s Medium (DMEM), supplemented with 10% fetal bovine serum (FBS) and 1% penicillin-streptomycin solution. Cells were supplied with fresh medium three times a week and passaged at 80% confluence.

### Coronary stents, application of an AMF and temperature measurement

Stainless steel stents (316L) with typical coronary stent dimensions of a diameter of 3.5 mm and a length of 14.5 mm were provided by Beijing MED Zenith Medical Scientific Co., Ltd. (Beijing, China), and were used in the present study in an expanded form.

A portable inductive heating device with a frequency of 300 kHz and an adjustable field intensity was provided by Shuangping Instrument Technology, Co., Ltd. (Shenzhen, China). The field generator consisted of an alternating current generator feeding the coil inductor. A copper-constantan thermocouple temperature probe (model IT-18; Physitemp Instruments, Inc., Clifton, NJ, USA) was utilized for the temperature measurements. The probe fibers were connected to a four-channel millivoltmeter (model XSOL-4; Beijing Kunlun Tianchen Instrument Technology, Co., Ltd., Beijing, China) and the data were collected every 6 sec by a personal computer (PC) with home-written software. Prior to each experiment, the thermocouple temperature probe was calibrated at 0 and 100°C.

### Inductive heating properties of the coronary stent under AMF

**Figure 1.**
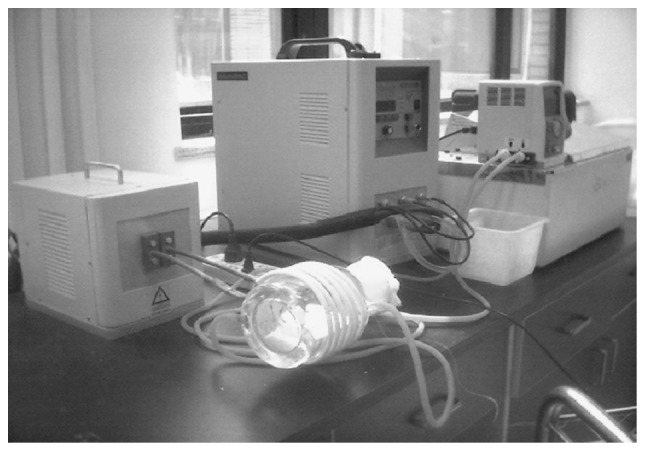
Portable inductive heating device and water jacket incubator.

The thermocouple probe was fixed at the surface of the stent in an expanded form by insertion into the mesh of the stent. Following this, the thermocouple-loaded stent was wrapped carefully in thermal insulation materials, such as asbestos fibers, and then placed into a water jacket incubator, which was designed for temperature maintenance. The double-layer jacket was connected to a water bath, so that it was possible to adjust and maintain the temperature inside the jacket at ∼37°C. The jacket was made of glass to prevent the device itself inducing heating when exposed to the AMF. Fig. 1 shows the experiment set-up for the evaluation of the inductive heating properties of the coronary stent.

### Microscopic observation of the cellular morphology of the VSMCs under MSH

**Figure 2.**
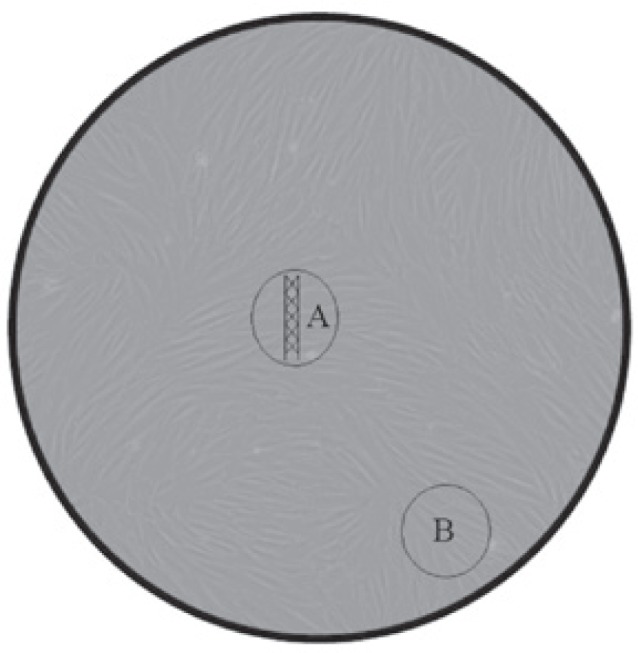
Illustration of the co-incubation of the stent and the vascular smooth muscle cells (VSMCs). (A) Stent-located area; (B) field of view outside the stent area.

Rabbit VSMCs were routinely cultured in the culture dish. When the cells reached 80% confluence, the stent was carefully attached to the cell monolayer with 2% agarose gel. The stent was co-incubated with the VSMCs for 3 to 5 days prior to the initiation of MSH treatment for 10 min at different treating temperatures. Following the MSH treatment, the cellular morphology of the VSMCs was observed under an inverted microscope. In order to examine the local heating effects produced by the MSH, two fields of view were selected for microscopic observation. These comprised one within the area of the stent location and one outside the stent area (at the edge of the culture dish). Fig. 2 illustrates the co-incubation of the stent and the VSMCs, and the two fields of view for microscopic observation. Subsequently, the cells were further cultured for up to 72 h to enable the analysis of cell proliferation, apoptosis and cell cycle, as well as to perform an immunohistochemical assay for PCNA expression.

### MTT assay for cell proliferation

The effect of the heat treatment at different temperatures was assessed using a colorimetric MTT assay, and compared with that in the control area. Following treatment, the cells were harvested with trypsin-ethylenediaminetetraacetic acid, seeded in 96-well microtiter plates at a density of 5,000 cells per well and incubated at 37°C in a humidified atmosphere with 5% CO_2_ for different durations. Subsequent to incubation, 20 *μ*l 10 mg/ml MTT solution was added to each well and the plates were incubated for 4 h, allowing the viable cells to reduce the yellow MTT into dark blue formazan crystals, which were dissolved in 150 *μ*l dimethyl sulfoxide (DMSO). The absorbance of the individual wells was measured at 490 nm using an automated microplate reader (Bio-Rad, Hercules, CA, USA).

### Annexin V-fluorescein isothiocyanate (FITC)/propidium iodide (PI) double-staining assays of the apoptotic cells

The occurrence of apoptosis and/or necrosis was evaluated by Annexin-V binding and PI uptake. Annexin-V binding was performed using an Annexin-V-FITC kit (Kaiji Co., Ltd., Nanjing, China), in accordance with the manufacturer’s instructions. Cells were plated at a density of 1×10^6^ cells/well into 24-well plates for 24 h and were pretreated with various concentrations of methanol extract (25 and 50 *μ*g/ml) of binding buffer. After 24 h, deoxyribose (dRib; 50 mM) was added to the plates, which were incubated at 37°C for an additional 24 h. The cells were then harvested, washed with phosphate-buffered saline (PBS) and suspended in 100 *μ*l Annexin-V binding buffer [containing 10 mM 4-(2-hydroxyethyl)-1-piperazineethanesulfonic acid (HEPES)/NaOH (pH 7.4), 140 mM NaCl and 2.5 mM CaCl_2_]. Following this, the cells were double stained with 10 *μ*l FITC-labeled Annexin-V and 10 *μ*l PI solution (containing 50 *μ*g/ml PBS). The samples were then incubated for 20 min at room temperature and analyzed using flow cytometry (BD Biosciences, Franklin Lakes, NJ, USA).

### Cell cycle analysis

The cells were harvested, washed with PBS, resuspended in 200 *μ*l PBS and fixed in 800 *μ*l iced 100% ethanol at −20°C. Having been left to stand overnight, cell pellets were collected by centrifugation, re-suspended in 1 ml hypotonic buffer (0.5% Triton X-100 in PBS and 0.5 *μ*g/ml RNase), and incubated at 37°C for 30 min. Following this, 1 ml PI solution (50 *μ*g/ml) was added, and the mixture was allowed to stand for ≥30 min at 37°C in the dark, prior to being filtered through a nylon mesh of 400 screen meshes. A total of 1×10^6^ cells were analyzed by a fluorescence-activated cell sorter caliber II (FACSCaliber II) cell sorter and the Cell Quest FACS system (BD Biosciences). The experiment was repeated three times and an average was taken from the three results. No less than 10,000 cells were analyzed in each sample. The percentages of cells in the G0/G1, S and G2/M phases were determined by the FACSCalibur II (BD Biosciences).

### Immunohistochemical localization of PCNA protein

PCNA immunodetection was conducted using a PCNA staining kit (ZSGB-Bio, Beijing, China) and the procedure was performed according to the protocol described by Raucci and Di Fiore (16). An anti-PCNA mouse antibody in a dilution of 1:100 and a goat anti-mouse immunoglobulin (Ig) G-horseradish peroxidase (HRP) antibody in a dilution of 1:200 were used. Cells were stained with the diaminobenzidine (DAB) substrate and visualized using a light microscope.

### Scratch wound healing assay

The effect of the heat treatment on the migration activities of VSMCs was assessed using a scratch healing assay, according to the protocol of Liang *et al* (17). Briefly, cells were seeded into 12-well cell culture plates for routine culture to produce a nearly-confluent cell monolayer. A linear wound was subsequently generated in the monolayer using a sterile 200 ml plastic pipette tip to produce an ∼1 mm-wide scratch. Any cellular debris was removed by washing the wells with PBS. Cells were then treated with different thermal doses by water-bath heating. Digital images were captured at 0, 4, 8, 18 and 24 h subsequent to the creation of the scratches to document the results.

### Statistical analysis

Results are expressed as the mean ± standard deviation (SD). Statistical analyses were performed using SPSS statistical software (SPSS, Inc., Chicago, IL, USA). Group comparisons were performed using the Student’s t-test followed by the least significant difference t-test (LSD-t). P<0.05 was considered to indicate a statistically significant difference.

## Results

### Inductive heating properties of the 316L stainless stent under an AMF

**Figure 3.**
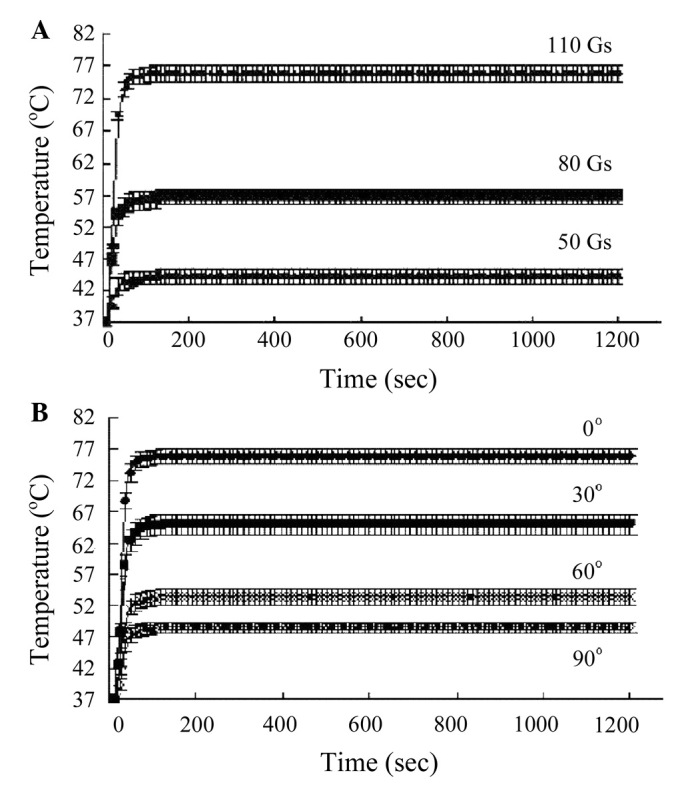
Inductive heating properties of the 316L stainless stent under alternative magnetic field (AMF) exposure. Effects of (A) field intensity and (B) orientation of the stent axis.

Fig. 3 demonstrates the inductive heating profile of the 316L stainless stent under AMF exposure. Fig. 3A shows that the 316L stainless steel stent possessed ideal inductive heating properties under AMF exposure with a frequency of 300 kHz. Rapid temperature increases, as shown by the initial slopes of the curves, were observed and the equilibrium temperature was reached within 100 sec. The equilibrium temperature was then maintained stably throughout the observation period. Fig. 3A also shows that field strength was directly correlated with the inductive heating characteristics of the stent, with a higher field strength resulting in a higher equilibrium temperature.

Fig. 3B shows the effect of the orientation of the stent axis on the inductive heating of the stent. The results demonstrate that a maximal temperature increase in the stent was achieved when the stent was positioned parallel to the field direction, i.e. with 0° angle between the stent axis and the direction of the AMF. In addition, the temperature increase was compromised by the angle increase, with a minimal temperature increase obtained at an angle of 90°.

### Microscopic observation of VSMC morphology under MSH

**Figure 4.**
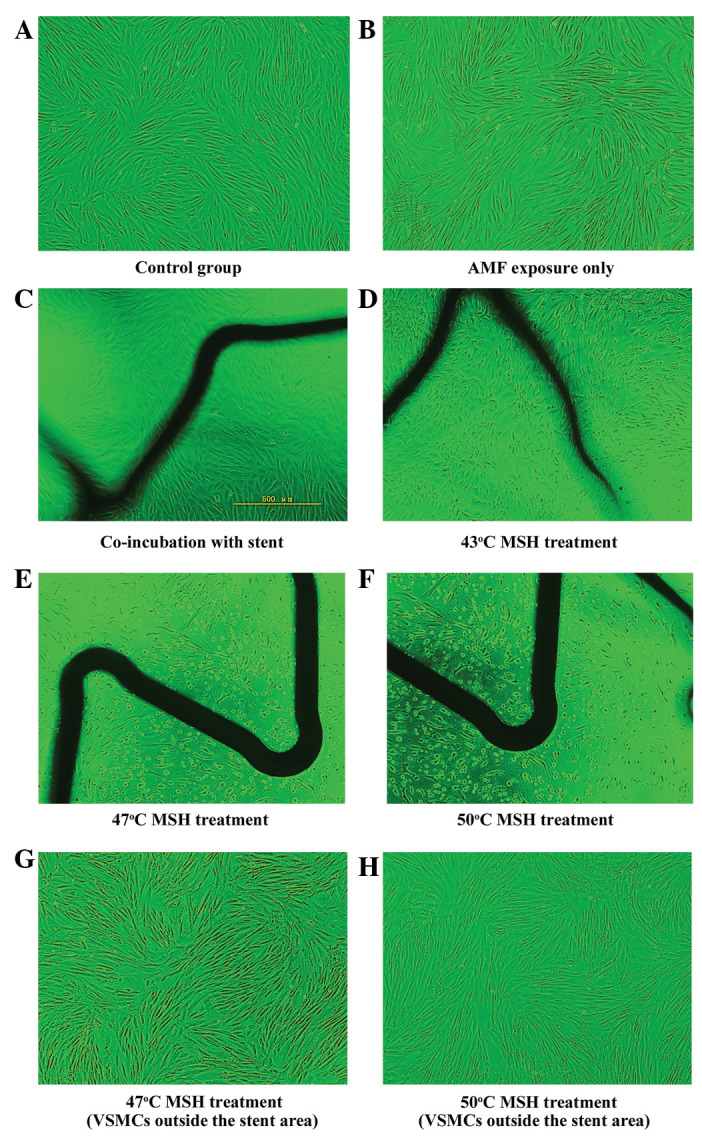
Effect of magnetic stent hyperthermia (MSH) on vascular smooth muscle cell (VSMC) morphology. Magnification, ×200.

Fig. 4 shows the microscopic observations of the VSMC morphology subsequent to MSH treatment for 10 min at different temperatures. Prior to MSH treatment, the cells were co-incubated with the stent for 3–5 days. As shown in Fig. 4A and C, no difference was observed between the VSMC cultures with or without the stent, indicating the biocompatibility of the stent. Fig. 4 also demonstrates that the shape and living status of VSMCs were markedly influenced by the MSH treatment, depending on the temperature and the location of the VSMCs. Although in the same culture dish as the stent, the morphology of the cells outside the stent area remained unaffected, and consistent with the cells in the control group, indicating that MSH produced a localized and specific effect. With regard to the cells cultured within the stent area, there were no marked changes in cell morphology following a 43°C MSH treatment, as Fig. 4 shows that the cells were spindle-shaped and well-arranged, indicating a good growth. However, following MSH treatments at ≥47°C, typical apoptotic morphological changes were observed. The VSMCs demonstrated a tendency to grow in a rounded shape, and adhered poorly to the culture dish. In addition, there were increases in the space between cells and in the numbers of free cell, as shown in Fig. 4E and F. It was also notable that the cells under AMF exposure only (without the stent) maintained the same morphology as those in the control group, suggesting that AMF exposure contributed little to the effect of MSH on cell morphology (Fig. 4B).

### Effect of MSH on cell proliferation

**Figure 5.**
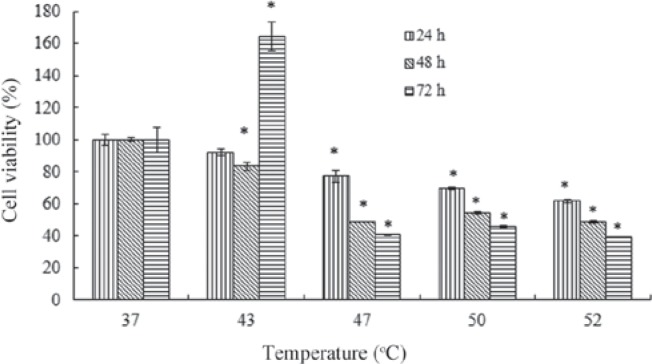
Effect of magnetic stent hyperthermia (MSH) on vascular smooth muscle cell (VSMC) proliferation (n=6). ^*^P<0.05 compared with the cells in the 37°C group.

Fig. 5 shows the dual effect of the heat treatment on the viability of the VSMCs. The effect was temperature- and incubation period-dependent. For 24 and 48 h incubation periods following heating, a 43°C treatment demonstrated almost no effect on the cell viability. However, treatments at ≥47°C resulted in reductions in cell viability to significantly greater extents. By contrast, there was a marked change in the antiproliferative effect of the 43°C heat treatment following a 72 h incubation period, as an increased viability was observed. This phenomenon only occurred with the 43°C heat treatment, and in the groups treated with temperatures ≥47°C there was a reduction in cell viability compared with the control group.

### Effect of MSH on cell apoptosis

**Figure 6.**
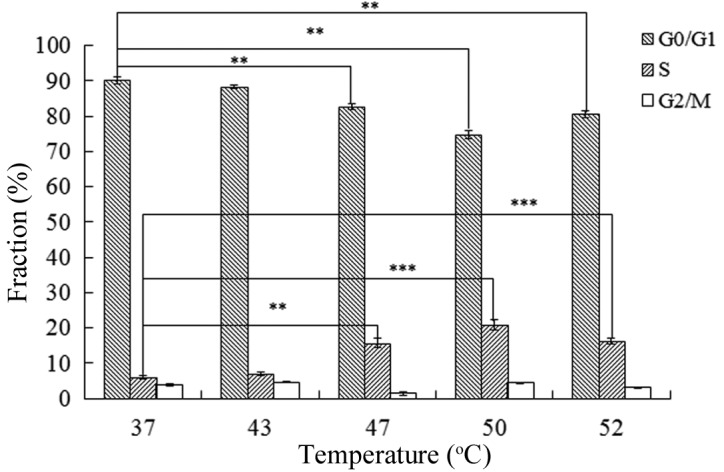
Effect of magnetic stent hyperthermia (MSH) on vascular smooth muscle cell (VSMC) apoptosis (n=6). ^**^P<0.01 and ^***^P<0.001.

As described in previously, Annexin V-FITC/PI double-staining assays were performed for the analysis of cell apoptosis. As shown in Fig. 6, following MSH treatments, the VSMCs exhibited a temperature-dependent increase in apoptosis. The increases in apoptosis with MSH treatments at ≥47°C were statistically significant when compared with control group. However, the 43°C MSH treatment was observed to have a negligible effect on the apoptosis of the VSMCs. These results indicated that MSH at ≥47°C effectively led to the apoptosis of the VSMCs. It was noted that AMF exposure demonstrated little effect on cell apoptosis. The results regarding the effect of MSH on apoptosis were consistent with the morphological observations, as well as the results from the cell proliferation analysis.

### Effect of MSH on the cell cycle

FACS analysis was used to investigate the effect of temperature on the cell cycle of the VSMCs. The percentages of the cell populations in the G0/G1, S and G2/M phases were 86.60, 2.10 and 9.76%, respectively, in the control group. Following MSH treatment at different temperatures, as shown in Table I, the cell populations in G0/G1 phase decreased, indicating S and G2/M phase arrest. There were statistically significant differences between the experimental and control groups, with the exception of MSH treatment at 43°C.

### Effect of MSH on PCNA expression

**Figure 7.**
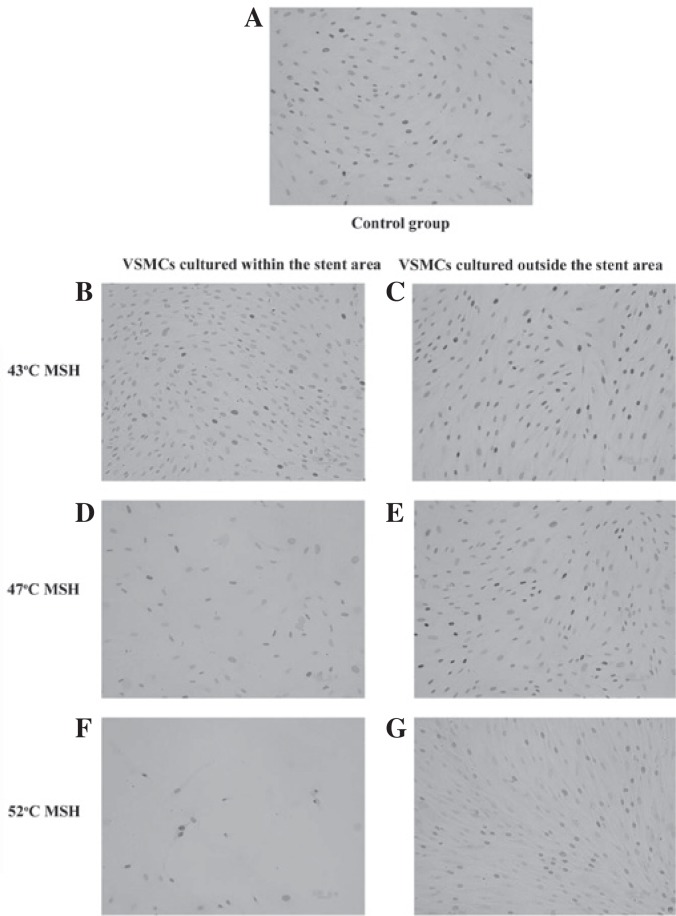
Effect of magnetic stent hyperthermia (MSH) on the proliferating cell nuclear antigen (PCNA) expression of vascular smooth muscle cells (VSMCs). Magnification, ×200. Cells were stained with the diaminobenzidine (DAB) substrate and visualized using a light microscope.

The reduction in the proliferation rate of VSMCs following MSH treatment at higher temperatures was further evaluated using immunohistochemistry to examine the expression levels of PCNA. Fig. 7 demonstrates that the expression level of PCNA was markedly inhibited by the MSH treatment, with more significant changes observed when the temperature was above 47°C. Following MSH treatment at 50°C, the expression of PCNA was reduced markedly, indicating that MSH treatment produced an anti-proliferative effect at higher temperatures. The results also demonstrated that, regardless of the temperature, the effect of MSH on PCNA expression was limited to the VSMCs cultured within the stent area, since the PCNA expression level in the cells outside the stent area was not markedly affected by the MSH, despite the cells being in the same culture dish as the stent. This result was consistent with the microscopic observations of the cellular morphology of the VSMCs, and further demonstrated the localized effect of the heat treatment.

### Effect of heat treatment on the migration activities of VSMCs

**Figure 8.**
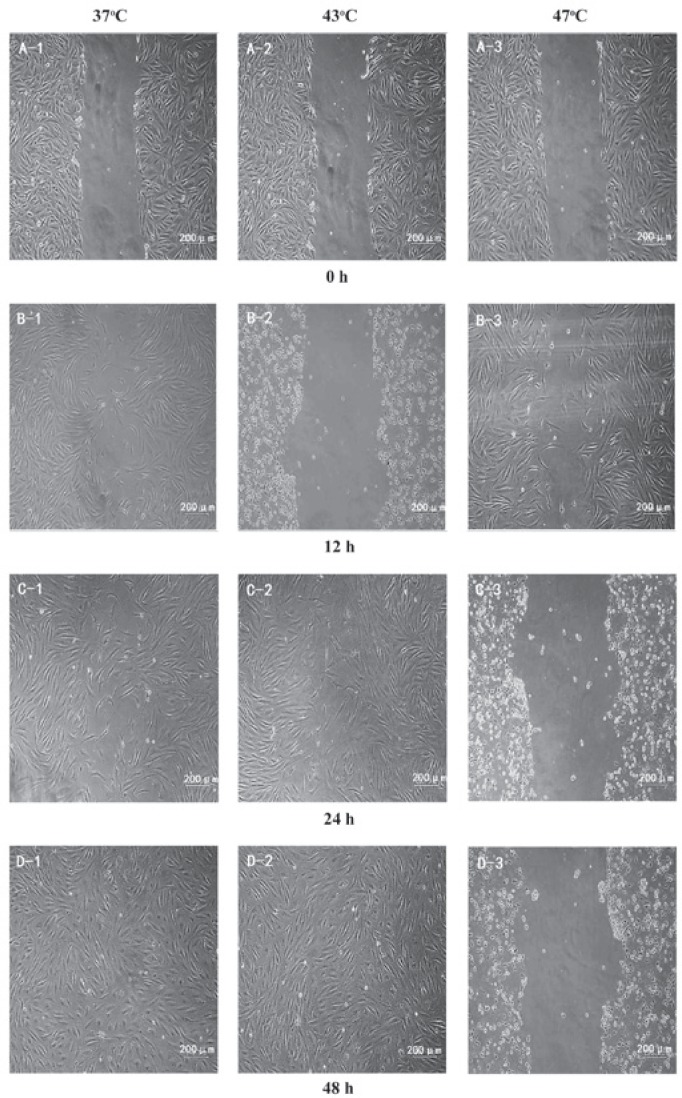
Effect of magnetic stent hyperthermia (MSH) on vascular smooth muscle cell (VSMC) migration. Magnification, ×200.

Fig. 8 shows the effect of the heat treatment at various temperatures on the migration activities of VSMCs, as observed using an *in vitro* scratch assay. In the control group, the cells gradually repopulated the cell-void area following scratching. A complete recovery of the scratch was obtained within 48 h. A 43°C heat treatment demonstrated similar results to those of the control group. Moreover, the cell density of the scratched area appeared to be higher than that of the control treatment area. However, the 47°C heat treatment resulted in a significant and long-term effect on VSMC migration. It was demonstrated that 48 h following the scratching, almost no cells had migrated to the void area.

## Discussion

Restenosis, or renarrowing or the arteries, is largely a result of the body’s own wound healing response to the mechanical injury that occurs with stent implantation. A greater understanding of the cellular processes involved in restenosis is required, although abnormal proliferation and migration have been suggested to be predominant features. At present, DES implantation is the most frequently adopted approach in the treatment of restenosis. DES implantation implements a controlled delivery system for a local, site-specific drug release for neointima formation; however, as mentioned previously, although the clinical and long-term outcomes of DES implantation have justified the role of DESs in curbing undesirable neointimal hyperplasia, risks concerning instent thrombosis have been identified. Therefore, an effective and safe technology based on BMSs is highly desired.

Although the beneficial effects of hyperthermia on the cardiovascular system have been gradually revealed, an unequivocal identification of the mechanisms leading to the favorable clinical results of hyperthermia have not yet been elucidated. The technical limitations of the locoregional delivery of heat and the poor control of the thermal dosage are possible factors impeding the successful application or translation of the research into clinical practice. The recent breakthrough in magnetic-mediated hyperthermia (MMH) may lead to the development of a novel alternative for locoregional hyperthermia, as it couples the heat magnetically to the mediators or agents within the target site only. Since the concept of MMH was first proposed by Gilchrist *et al* (18) in the 1960s, following years of investigation, the developments in MMH have been successfully applied in clinical oncology with desirable results (18,19). However, little attempt has been made to expand the novel hyperthermia approach to other diseases, particularly cardiovascular disease (20). In the current study, the inductive heating property of a 316L stainless steel stent revealed that the expanded stent possessed ideal inductive heating characteristics upon exposure to the AMF. The parameters of the AMF, as well as the orientation of the stent inside the AMF, demonstrated significant effects on the stent’s heating characteristics. The ideal inductive heating properties of the coronary stent indicate the feasibility of the use of MSH in the treatment or prevention of restenosis.

As the mediator of MMH, the magnetic agents are critical in the hyperthermia treatment. Although the desirable inductive heating properties of the stent were demonstrated in the present study, it is necessary to note that the analysis presented in the study was a conservative one, due to the fact that it was in the absence of blood perfusion. In order to further improve the heating properties of the stent, Oya *et al* (21) developed a stent of magnetic shunt steel, excited by AMF, for thermo-therapeutic applications (21). Floren *et al* (20) proposed that it was possible to obtain enhanced heating results by coating the stent surface with ferromagnetic nanoparticles (20). Moreover, in recognition of the fact that temperature self-regulation has been fully achieved in MMH for cancer treatment by alloy thermoseeds with specific Curie-points, a coronary stent with the appropriate Curie point is highly desired for MSH.

For hyperthermia treatment, the therapeutic effectiveness is closely associated with the temperature during the treatment. The current study demonstrated that heat treatment exhibited an effect on the proliferation and migration of VSMCs, and that such effect was temperature-dependent. In general, a 43°C treatment demonstrated little effect on cell morphology, and did not effect cell proliferation or migration. However, an abnormal induction of VSMC proliferation was observed following 72 h incubation with 43°C heat treatment. Higher temperatures were demonstrated to exert a significant antiproliferative effect on the VSMCs and a marked inhibitory effect on cell migration. Brasselet *et al* (22) studied the effect of localized heating by *in situ* heated water on restenosis in a rabbit model. The results demonstrated that treatment at 50°C reduced instent neointimal hyperplasia, without the induction of thrombosis (22). The conclusions of the present study were consistent with the observations made by Li *et al*, in a study investigating the effect of heat treatment on the shape and living status of VSMCs (23). The results were categorized into three stages: i) At temperatures <44°C, the living status was not changed; ii) between 44 and 50°C, the cells demonstrated shrinkage, but remained alive; iii) at temperatures >50°C, all cells died. Although the present results showed that some VSMCs remained alive following MSH treatment at 50°C, the present study and that by Li *et al* concurred with regard to the conclusions that the effect of hyperthermia was temperature-dependent, and that temperatures <43°C demonstrated no effect on cell growth. The difference between the two investigations may be due to the difference between the two heating approaches, as the present study used MSH while the study by Li *et al* used water bath heating. Since the proliferation and migration of VSMCs mainly account for the restenosis, there is a requirement for higher temperatures to be considered for MSH in the treatment of restenosis.

The current study investigated the possible mechanisms behind the VSMC proliferation following MSH treatment through the measurements of PCNA expression and the inhibition of cell cycle progression. The results from the cell cycle analysis demonstrated that there was significantly less progression to the G0/G1 phase in MSH-treated cells than in the control group 24 h following the heat treatment, suggesting S and G2/M phase arrest. Orihara *et al* (15) evaluated the effect of heating on VSMCs from the rat thoracic aorta and revealed that the results indicated G1 arrest (15). Two plausible explanations may account for the difference between the two observations: There was an interspecies difference between the two studies and the two heating approaches, i.e. MSH or water bath heating, may have had an effect. Immunochemistry was used to study the effect of MSH on PCNA expression, with the results clearly demonstrating that MSH exerted a significant effect on the PCNA expression of the VSMCs. The higher the temperature, the lower the level of PCNA expression observed. PCNA is a protein that acts as a processivity factor for DNA polymerase δ in eukaryotic cells, and was originally identified as an antigen expressed in the nuclei of cells during the DNA synthesis phase of the cell cycle. PCNA is important for DNA synthesis and repair. The reduced expression of PCNA following MSH treatment may explain the inhibition of the VSMC proliferation.

An important issue to be considered with magnetic hyperthermia is the contribution of the AMF exposure to hyperthermic cytotoxicity. At present, the field frequency of AMF for magnetic hyperthermia is of an intermediate frequency range. The field effects of frequencies ≤1 kHz and >1 MHz are well known; however, the intermediate frequency range lacks intensive investigation and, to date, has received little attention. The current investigation showed that AMF exposure had a negligible effect on the growth status, proliferation or apoptosis of the VSMCs. This observation was consistent with our previous study, in which we demonstrated that 100 kHz AMF exposure had little effect on the proliferation and apoptosis of human esophageal cancer cells (24). The present results suggested that AMF exposure (in the intermediate frequency range) contributed little to the effect of magnetic hyperthermia.

One unique favorable feature of MSH is that it specifically heats only the target site loaded or infused with magnetic mediator. The present study provided results that demonstrated the localized heating effect of MSH. Even in the same culture dish, the apoptotic morphological changes and the inhibition of the PCNA expression only occurred to the cells cultured in the proximity of the stent, while cells outside the stent area were not affected. Therefore, it may be concluded that MSH heating is restricted within the stent location and thus only induces a small or even negligible injury to the nearby tissues during the treatment.

From the previously mentioned discussions, it may be concluded that the thermal effect on the growth of VSMCs was temperature-, cell line- and heating approach-dependent. In order to provide a more direct insight into the effect of hyperthermia on the prevention and treatment of restenosis for clinical application, investigations involving human VSMCs (hVSMCs) are required. In addition, further systematic studies are required to address the different effects of the two different heating approaches on cell growth and to optimize the treatment time and temperatures. Such studies are under close investigation in our laboratory.

In conclusion, the results of the present study demonstrate that 316L stainless steel stents possess ideal inductive heating characteristics under AMF exposure for clinical application. MSH treatment has significant effects on the proliferation, apoptosis and migration of VSMCs, and the effects of MSH are temperature-dependent. MSH treatment at temperatures >47°C effectively inhibits the proliferation and migration of VSMCs. The possible mechanisms behind the inhibition of proliferation by MSH may be due to a reduction in the progression to the G0/G1 phase of the cell cycle, in addition to the inhibition of PCNA expression.

## Figures and Tables

**Table I.** Effect of MSH on the cell cycle of VSMCs (n=3).

Treatment	Cell cycle phase		

	G0/G1	S	G2/M
Control	86.607±0.246	2.100±0.148	9.760±0.056
43°C	85.813±0.831^a^	2.730±0.380	9.987±0.100
47°C	83.880±0.203^b^	2.803±0.188^a^	12.310±0.182^b^
50°C	71.620±0.726^b^	6.857±0.455^b^	16.397±0.430^b^
52°C	66.573±0.514^b^	8.650±0.128^b^	19.807±0.585^b^

a P<0.05 and

b P<0.01 compared with the control. MSH, magnetic stent hyperthermia; VSMC, vascular smooth muscle cell.
